# Pivoting in the pandemic: a qualitative study of child and adolescent psychiatrists in the times of COVID-19

**DOI:** 10.21203/rs.3.rs-287057/v1

**Published:** 2021-03-04

**Authors:** Madeline DiGiovanni, Indigo Weller, Andres Martin

**Affiliations:** Yale School of Medicine: Yale University School of Medicine; Harvard University; Yale University School of Medicine

**Keywords:** qualitative methods, COVID-19, advocacy, telemedicine, semi-structured interviews

## Abstract

**Objectives:**

We examined the personal and professional impacts of the COVID-19 pandemic on the development, practice, and shifting values of child and adolescent psychiatrists (CAP), in order to inform how the field may move forward post-pandemic.

**Methods:**

We conducted individual semi-structured interviews of child and adolescent psychiatrists (n = 24) practicing in the US. Participants were selected as a diverse purposive sample of active members of the American Academy of Child and Adolescent Psychiatry (AACAP). We analyzed anonymized transcripts through iterative coding using thematic analysis aided by NVivo software.

**Results:**

We identified three main thematic domains within participants’ response to the pandemic, which have engendered a reevaluation of and a recommitment to the aims of each clinician and the field of CAP more broadly. These domains, paired with representative questions, include: 1) *Unsettling*, or “who have we been?” (identifying discontents such as daily inefficiencies and intraprofessional loss of trust); 2) *Adaptation*, or “who are we now?” (exploring affordances and limitations of virtual work, and the evolution of personal and professional identity); and 3) *Reimagination*, or “who will we become?” (renewing a commitment to psychiatry as advocacy). Even as we identified a collective agreement toward the need for implementing change, just what needs to change, and how that change will be realized, remain contested.

**Conclusion:**

These three thematic domains, augmented by a national confrontation with race and equity, have engendered a field-wide reckoning with known inequities. They have reinvigorated collective responses and calls to action. The divergent mindsets to change and leadership have provided an aperture for what values and practices the field might instill in its next generation of practitioners.

## Background

With the COVID-19 pandemic’s arrival in the Unites States in early 2020 [1], healthcare workers faced a rapidly evolving set of novel demands and an uncertain course. Emergence of the “healthcare hero” narrative highlighted health professionals’ role as acute responders to the national crisis, a responsibility accompanied by significant mental health concerns as the challenges of the pandemic compounded. In one US study, healthcare providers reported higher levels of depressive symptoms, current anxiety, and COVID-19-related stress, as well as lower levels of perceived control and proactive coping, compared to non-healthcare providers [2], findings that echo similar studies showing increased COVID-19-related distress among healthcare workers in multiple other countries [3–6]. Sources of distress include shortages of personal protective equipment (PPE), concerns about COVID-19 morbidity or mortality for oneself and others, fear of bringing the virus home to family, and lack of up-to-date information or effective communication, among other concerns [7], illustrating an emerging tension between the venerated “hero” role and the often-lacking support for frontline providers to perform their duty safely.

Frontline healthcare providers are not the only ones who experienced the negative mental health consequences of the pandemic. As medicine moved quickly to address the infectious disease crisis, a slower-burning but increasingly urgent mental health toll began mounting for the general public. By mid-2020, American adults reported increased levels of depression, anxiety, substance use, and suicidal ideation [8], alongside significant shifts in how mental health providers could offer care given the restrictions of the pandemic [9]. Children and adolescents were one of several populations disproportionately affected by the mental health toll of the pandemic [8], given the increase in adverse childhood experiences such as child abuse, neglect, and intimate partner violence [10–12], and the loss of protective factors such as schools, a significant mental health resource for many American children [13, 14]. Social distancing requirements also contributed to loneliness, a factor implicated in one study’s report that when compared to adults, US adolescents were significantly more likely to report elevated depression, anxiety, suicidal ideation, and sleep problems [15]. The increased mental health consequences for children and adolescents are already manifesting changes in mental healthcare usage: Despite fluctuations in children’s mental health-related emergency department (ED) usage, the proportion of all ED visits for children’s mental health–related concerns increased and remained elevated, suggesting that children’s mental health concerns were significant enough to warrant emergent visits during a time when most others avoided the ED if possible [16].

Child and adolescent psychiatrists (CAPs) occupy a unique role in managing the psychosocial consequences of the pandemic and in shaping the country’s emergence from crisis. As healthcare providers themselves, many CAPs are at risk for the same COVID-19 stressors plaguing providers across medicine. Simultaneously, they shoulder the responsibility of supporting the increased psychiatric needs of children and adolescents amidst an ongoing national crisis. With the arrival of new COVID-19 strains and the shaky rollout of the US vaccination protocol [17, 18], the challenges of the pandemic are not likely to abate soon, leaving CAPs (among other leaders in medicine) with the additional task of determining what strategic next steps are necessary to minimize long-term negative impact on child mental health.

Yet the COVID-19 pandemic is not the only crisis confronting leaders in CAP and medicine more broadly. As the virus was taking hold of the country, the United States began to grapple with a renewed confrontation with systemic racism. By April 2020, data emerged illustrating the disproportionate racial impact of COVID-19 on Black Americans [19], reinvigorating wider conversations on social determinants of health and medicine’s complicity in racism. By midyear, the nationwide reckoning intensified more publicly with a surge in Black Lives Matter (BLM) protests, in part sparked by the unjust killings of George Floyd, Breonna Taylor, Ahmaud Arbery, Rayshard Brooks, Tony McDade, and others throughout the year. America faced not just a pandemic, but a syndemic. The term *syndemic*, coined by American medical anthropologist Merrill Singer in his work on the AIDS epidemic [20], represents the synergistic interaction of health and social crises to increase risk of negative health outcomes for specific populations. In the US context, the synergistic crises of COVID-19 and systemic racism manifested in a syndemic that disproportionately impacted BIPOC (Black, Indigenous, and People of Color) communities [21, 22].

In the face of this confluence of crises, leaders within CAP and medicine bear a responsibility not just to mitigate the harm of COVID-19 on their patient populations, but also to address the role of racism in their specialties and implement practices to address racial disparities [23, 24]. Multiple medical organizations have released statements committing to combat racism, and many medical schools or institutions have seen an increase in anti-racist protests like #WhiteCoats4BlackLives; but it will be the concrete actions and priorities of medical leadership that determine each specialty’s future track record on systemic racism. Balancing the competing urgencies of COVID-19 and systemic racism requires effective adaptation and leadership, and we hope that examining the current responses of CAP providers and leaders to the syndemic can provide a window into how the field can most effectively move forward post-pandemic.

In a recent paper from our group, Herrington et al [25] explored how mental health providers working with children and families around the globe responded to COVID-19 by co-creating a “viral time capsule” of the early months of the pandemic.

The authors wrote that it “will be contingent on us, whether as a field we will rise to the occasion and capitalize on the opportunity to improve mental health services that the pandemic crisis has abruptly opened.” In this paper, we seek to identify the priorities and mechanisms for how exactly CAPs in the US plan to rise to the occasion, to inform how leadership can efficiently guide the field forward post-pandemic.

Additionally, the authors touched on the existence of disparities in resources and outcomes on a global scale. They argued that any global account must first “provincialize” the American experience with racism to resist framing the global pandemic from a solely Western context [25]. In response, our paper focuses explicitly on the US context and seeks to articulate the array of challenges and opportunities facing the field of CAP amidst the uniquely American syndemic experience [26].

Whereas Herrington et al provided the “camera” to take time capsule “snapshots” through photo-elicitation methods, in this study we aim to “develop” the photographs: to bring specific dynamics into focus, to explore the apertures for and exposures of certain viewpoints, and to highlight the process of change in order to visualize a narrative arc of how the field of CAP can effectively move forward.

To that end, we used individual, in-depth, semi-structured interviews, in which each participant offered the concept of “pivot points”: the shifting of existing momentum in a new direction.

The field currently juggles the energies of competing crises, and how and where the field chooses to pivot dictates where future momentum is directed. By exploring the early shifts and pivots of CAPs in the context of the US, we aim to apply lessons learned about change, leadership, and development within CAP to identify the field’s next steps in service of more equitable child and adolescent mental health.

## Methods

### Design

We conducted individual, semi-structured interviews, complemented by a small photo-elicitation component, in which participants submitted a set of photographs representing their life and practice during COVID-19 along with a reflective exercise around a prepared list of sensitizing questions (Appendix 1). Semi-structured interviewing is a flexible, commonly used method in qualitative research in healthcare [27] that utilizes a prepared list of questions to guide researchers and participants to “co-create meaning” through an exploration of participants’ opinions and insights, especially for potentially sensitive or personal topics [28, 29]. This method’s focus on “reconstructing perceptions” [28] pairs well with photo-elicitation, which originates from American anthropologist John Collier and incorporates photographs into a research interview to provide a “restatement of reality” to the participant [30]. Photo-elicitation has traditionally been used in sociology and anthropology [31], with more recent inclusion in healthcare and psychology [25, 32–35]. The approach offers the additional benefit of centering the participant’s frame of reference, thereby diminishing the researcher’s implicit “agenda” [36].

### Participants and ethics approval

We selected participants as a purposive sample of active members of the American Academic of Child and Adolescent Psychiatry (AACCAP). In recruiting participants, we were attentive to include varied representation across gender, career stage, geographic region, racial identity, ethnicity, and professional practice setting (Appendix 2). We obtained ethics approval from the Yale University Institutional Review Board (Protocol #2000028102).

Our study was deemed exempt under 45CFR46.104 (2)(ii) and did not require participants’ written informed consent.

All results exclude any potentially identifying information.

### Data collection

Prior to each interview, we invited the participant to submit a series of photographs with no minimum or maximum number (suggested 3–5), with the option of an additional written or voice-recorded reflection. We sent invitations via email from April to August of 2020, using the language below:

We invite you to participate in a reflective exercise that uses photography followed by a semi-structured interview to explore how COVID-19 may have shifted your personal and professional life as a child psychiatrist. This exercise will serve as the basis for a qualitative research study that aims to identify the present benefits and common challenges to well-being and mental health for practitioners like yourself. More specifically, our goal is to better understand how these changes to clinical practice, teaching, and leadership amidst this period of prolonged uncertainty may have affected your sense of well-being and coping resources, shifted work-life balance, and blurred the boundaries between your personal and professional roles.

Our invitation also included the list of sensitizing questions and a sample submission.

Each interview was transcribed for analysis to create our final data set, with the participant photographs and written reflections serving as a reflective exercise rather than as analyzed data. All but four participants submitted images, with one of the four presenting a physical object during the interview in lieu of a photograph. Thirteen participants accompanied their photographs with written reflections. We uploaded transcripts into qualitative analysis software (NVivo version 12; QSR International, Melbourne, Australia), adding each participant’s contributions throughout the interview months to ensure that analysis remained an iterative process.

### Data analysis

We coded the transcripts according to the principles of thematic analysis, a qualitative approach involving the active construction of overarching patterns and meaning across a dataset [37, 38]. Thematic analysis has been previously paired with photo-elicitation in adolescent psychiatry [25, 34], and it allows for rich, flexible exploration of the data to construct themes that “reframe, reinterpret, and/or connect elements of the data” without developing a final theory, as does grounded theory [38]. In this project, we followed Braun and Clarke’s six-step method and adopted a more inductive than theoretical analytic approach, allowing our research questions to evolve beyond our sensitizing questions as both the cultural context and the content of the interviews changed over time [37].

Two authors (MD, IW) coded independently throughout the study span in an iterative manner, with coding and interviewing interdigitated to allow for the inductive approach to shape subsequent interviews. The authors combined codes and assessed the final codebook to eliminate redundancies, clarify domains, themes, and codes, and ensure theoretical sufficiency [39]. Each final code was supported by quotes from more than one participant.

## Results

### *Unsettling:* Who have we been?

The COVID-19 pandemic catalyzed significant disruptions to the status quo, serving as a “wake-up call” that exposed dissatisfactions and disappointments for most participants. We identified the first domain, “Unsettling,” as asking the question: *who have we been*? Participants described exposures of the pandemic that served to “shine a light on” or “illuminate the underbelly” of three key areas of dissatisfaction: 1) daily workplace inefficiencies; 2) barriers to patient care; and 3) intraprofessional devaluation and loss of trust.

#### Daily workplace inefficiencies

As workplaces implemented social distancing and other public health precautions, many participants experienced a rapid migration to remote work and telehealth, catalyzing a reevaluation of prior in-person practices. In response to these shifts, participants spoke of a desired “rebalancing of what’s important [and] how we use our time” to achieve a streamlined, more efficient practice, given the affordances of virtual work. For example, a primary complaint of pre-COVID-19 times was “useless” meetings and “fluff time,” which participants identified as ineffective and bureaucratic. By contrast, some found virtual meetings more efficient in certain ways: “People are not commuting. People are not chitchatting. People are not wasting as much time.” Participants also desired a greater sense of urgency, frustrated by what they identified as excessive steps in research study onboarding and operational change in pre-COVID-19 protocols. Energized by the swift rollout of revised telehealth regulations and licensing changes, participants hoped for similar implementation of improved telehealth practices beyond COVID-19: “We can’t just follow our usual process…and have it take a month or two to implement. We have to do this *today*.”

In this way, the pandemic acted as a galvanizing force for reevaluating competing urgencies, with some arguing that COVID-19 “imposed the need, at least temporarily” for resolving ineffective practices to encourage swifter responses to current barriers. Specifically, participants highlighted the inefficiencies surrounding existing needs that had been previously deprioritized: “The urgency that the pandemic has created has allowed a lot of institutions and entities to respond to needs in a way that they were not responding before, even when you could say that those needs were present already.” Others framed the pandemic as “not a time for lip service” but rather to engage in more collaborative action to refine outdated practices to advance the field and its service to patients.

#### Barriers to patient care

The migration to telehealth also catalyzed shifts in patient access to care, highlighting and addressing several overarching logistic and financial barriers that participants had struggled with pre-pandemic. Participants commonly marveled how “suddenly we can deliver care where there hadn’t been any available means of access before.” Specific barriers included a dearth of local providers, transportation concerns, regulatory and insurance hurdles, and family dynamics. Telemedicine also revealed new concerns, such as disproportionate access to technology, both for telehealth and for education: “Some kids have access to the internet and some don’t. It’s not fair to punish those kids who don’t have access compared to the ones who do.” Additionally, children of essential workers experienced a new strain on parental availability for appointments. These barriers placed a renewed emphasis on addressing social determinants of health as a vital feature of basic advocacy within psychiatry: “If we don’t address those needs at the individual level, talking about really cool evidence-based therapy misses the mark.”

The opportunities for advocacy, long acknowledged by most, became pressing for many through growing recognition of the *syndemic*. Discussion of COVID-19’s racially disproportionate impact had been ongoing. However, for many participants the death of George Floyd and the subsequent surge in Black Lives Matter protests across the country mobilized an acute urgency and visibility to anti-racist work within the field. Participants spoke of the “layering of the two pandemics” and recognized systemic racism as a “public health crisis” that psychiatrists, as advocates, must address. One participant included a photograph of a protest they attended, where a demonstrator wielded a sign reading “racism *is* the pandemic,” noting that “I thought it was really important to have a picture that also is a timestamp to the uprisings and the way that they impacted not only the U.S. but other nations and the global impact that it had.”

Renewed confrontation of systemic racism’s place within child psychiatry specifically prompted several participants to look inwards to their own workplaces. One participant committed to investigating the disproportionate physical restraints for Black and Latinx children on their unit, and another described a deeper dive into existing data that illustrated a tenfold difference in involuntary procedures by school resource officers for Black students in the area. Like these two participants, others spoke of their ongoing and renewed commitment to psychiatry as advocacy, noting how their professional status affords them the knowledge, resources, and duty to fight for those with less representation. Several participants plainly stated that child psychiatry has not fulfilled this duty as effectively as colleagues in pediatrics: “Pediatricians have taken that mantle more aggressively and effectively than child psychiatrists.” To ensure CAP’s fulfillment of its responsibility, participants identified the need for collaboration with pediatricians and other providers, as well as interaction with insurance companies and local legislators to ensure that child wellbeing is supported by multiple stakeholders.

#### Intraprofessional devaluation and loss of trust

Adjustment to the new pandemic workplace created friction between colleagues and highlighted the shifts in intraprofessional relationships. These shifts made legible the long-standing divisions across departments and in turn revealed a tacit metric of professional value. Early administrative choices created conflict as some departments were deprioritized for PPE, left off key leadership emails, or given conflicting instructions about confidentiality protocol. Participants also frequently touched on a “differential of sacrifice” within departmental workforces. Given the complexity of scheduling in-person versus virtual shifts in an equitable way, they described a “level of entitlement” not previously seen. In one department, attendings shouldered the initial burden of in-person care to shield their inpatient team from early risk, only to receive a startling lack of reciprocal investment from team members. Some described colleagues as reluctant to risk the danger of working in person without being seen as “abandoning ship during a crisis.” As one participant from a hard-hit urban hospital system narrated:

“It was pretty evident very early on who took the attitude of, ‘Let’s jump in there. Let’s do everything we can to help. Let’s be on site, let’s be present. Let’s battle.’ And others who are like, ‘Get me out of here. I’m really concerned about myself. I’m really concerned about a family member. I’m really concerned about my kid. I want to be as far away, and as safe as possible.’”

The discrepancy in investment created disappointment and a loss of trust; but as one participant stated, “sunlight is a great disinfectant.” The revelation of previously hidden conflicts allowed space for healing and the need for “mutual sacrifices” among colleagues when distributing the workload to soothe rifts and “remain unified collectively.” Workplace rifts were common but not universal: several participants emphasized gratitude for their colleagues’ commitment and hard work. However, the wide expressions of gratitude were bound to a degree of disenchantment with psychiatry, finding an unsatisfying answer to the “who have we been?” question.

Some discontent stemmed from healthcare hierarchies in general, laid bare by pandemic decision-making and discussions of equity in medicine; and some were more specific to psychiatry, such as the “reactive-not-proactive” status quo (“Why aren’t we doing the outreach instead of waiting for people to come to us in crisis?”). Others reflected on psychiatry’s failures in social justice, limited training perspectives, shifting work values of trainees, and the manner of compensation that hampers effective collaboration. This discontent diminished some participants’ enjoyment of their careers and even triggered a reevaluation of one’s place in the field entirely, with one participant “questioning whether even my involvement in [professional organizations] is the best way to be using my resources.” Another noted that after two weeks of telemedicine, “I was just like, ‘I just want to do my laundry. I don’t want to be a psychiatrist today.’” Some identified this disenchantment as a pre-COVID-19 phenomenon, stating that “we’ve clearly been requiring a revolution in terms of medicine for a long time… and that has nothing to do with COVID-19 in my view.”

### *Adaptation:* Who are we now?

In response to these shifts and exposures, participants reported a wide range of adaptations to their personal and professional lives. We identified the second domain, “Adaptations,” as asking the question *who are we now*?, and is answered in three themes: 1) operational adjustments; 2) the affordances and limitations of virtual work; and 3) the evolution of professional and personal identity.

#### Operational adjustments

Like most sectors, the COVID-19 workplace upended daily norms and protocols for most participants. Even for those not working from home, physical workspaces evolved to include utilizing open-air playgrounds for inpatient viral testing, driving out to homeless populations instead of hosting indoor clinics, and holding telehealth appointments in cars between childcare duties. Inpatient unit censuses dropped for multiple participants, even leading to an accelerated closure of child services in one location during a building transition. Masks and other PPE created communication barriers: foggy goggles, muffled voices, and obscured facial expressions. At times, PPE had to be altered for certain patients, such as using face shields for children who could not tolerate masks.

For those who did switch to work-from-home, delineating the workday from home life (or not) created a new operational challenge. Participants reflected on the “occupational-residential merge,” manifested as converted office bedrooms or living among stacks of patient files, as well as the challenge of balancing a professional home workspace with family needs: spouses also working from home, students completing online school, and young children requiring supervision. Those who previously maintained stricter work-home boundaries depicted their divides as increasingly “permeable” and “frayed at the edges.” Some leaned into the dissolution of boundaries by being more available to patients after hours or emailing late into the night, while others sought to underline the end of the workday: keeping the laptop shut, creating an end-of-day dog-walking routine, or using a downtime app to limit evening phone use.

Regardless of their in-person or virtual status, most participants reported grappling with novel demands: “It turns out COVID-19 didn’t take away any of my responsibilities… It just added a couple jobs on top.” Directors navigated pay cuts, safety planning, and newly-limited inpatient capacity to prevent infectious spread. Virtual meetings were scheduled for early mornings, late nights, and weekends. Collaborations evolved both within the profession, such as increased participation in professional listservs or international projects, and across specialties, such as improved ER virtual consults. Participants also adapted to new educational demands. Across multiple institutions, training directors altered orientation, team-building, and graduation exercises to meet the needs of their now-virtual cohorts, incorporating breakout rooms for presentations and virtual diploma hand-offs; and medical school deans became “Grand Central Station for questions” for anxious medical students worrying about how their education could adapt to a pandemic.

Despite these increased educational demands, multiple training directors and educators savored the new opportunities for connection with trainees. In tele-adapted events, online platforms provided different yet equally meaningful ways to engage personally. One participant celebrated the new ability to engage more members of the trainee’s world, citing a mixed in-person/online graduation ceremony in which a small group of trainees and family members enjoyed an in-person celebration, followed by food delivered to each family’s location for a socially distanced meal and a “big Zoom graduation with 80 or 90 people from around the world that happened a couple of hours later.” Echoing the unexpected joy of a more intimate ceremony, another participant described how their Zoom graduation allowed the space for family members to share stories about their graduates during the ceremony, making the event “one of the most meaningful graduations I have ever been a part of.”

#### Affordances and limitations of virtual work

Beyond requiring adaptations to virtual workdays and training, telehealth afforded alternative avenues for communication and care. Virtual visits increased accessibility and decreased no-show rates, with one participant celebrating the marked decline in no-shows for mothers with post-partum depression given the lower activation energy necessary for session attendance: “Before… they just couldn’t get it together to come.” Participants also reported increased family member engagement and valuable insights into the patient’s home environment. Video backgrounds offered a novel source of contextual information, such as pets or artwork, which provided “meaningful connections to who they are as a person, which may not have come out in an in-person visit”. As for participants’ own backgrounds, most maintained firm boundaries with patients: “It’s clear that it’s my home…but I have really tried to maintain that it’s not overly personal.”

The affordances of increased connectivity, however, became a double-edged sword. Participants reported fatigue from back-to-back meetings, the intense focus of videoconferencing, and the constant flood of emails encouraged by a nonstop virtual workday. Put simply, “there was no turning off.” Participants also worried about the privacy afforded and the suspicion aroused by virtual backgrounds, along with the implications of pet or family member interruptions for professionalism, and the need for new boundary-setting with patients given the visibility of clinician homes or the settings of virtual care (e.g. addressing personal questions, patients attending sessions while driving). Some participants opted to continue going into the office for virtual visits to address these concerns. Increased visibility also necessitated a “different set of parameters” for acute intervention in the event of visible emergencies on camera, and the benefits of telehealth only extended to families with adequate internet connectivity and access to private spaces. Some mused about the long-term efficacy of telehealth, noting missing “emotional engagement” with some patients and not wanting to “settle” for a lower level of care in the future: “Tele is not a substitute for in-person visits. It’s an alternate treatment modality, but where exactly does it fit?”

The substance of each session adjusted to pandemic life, in both content and modality, depending on age group: “Early childhood mental health is a very physical kind of sport, and those tools are all out the window with Zoom.” Participants reported increased reliance on parental involvement for younger children: requesting pre-session email updates, focusing on the dyad, or leaning on virtual play and parental coaching. Discussions with older children highlighted struggles like concern for loved ones, others’ failure to social distance, and social development, including confusion about quarantine friendship protocols: “Are you on the level where you can FaceTime? Do you have those privileges?”

Most benefits of virtual care did not extend to virtual school collaborations in this study. Apart from one district that reworked an existing mental health curriculum for COVID-19 and grief coping, the closure of schools and school-based care hindered –and in some cases eliminated– child mental health care. Participants reported decreased interaction with teachers, perhaps due to reduced behaviors due to the different environment or increased direct communication with parents (“I cannot remember having spoken to any teacher since COVID-19 happened”) and a loss of special education services. Adherence to Individualized Education Programs (IEPs) suffered, given the difficulties of virtual school, and students who are nonverbal or with significant developmental disabilities were left without the specialists or much-needed services that special education programs provide. For all children, the attentional demands of virtual school were an unavoidable challenge: “It’s torture for an adult; just imagine for a kid.”

#### Evolution of personal and professional identity

In metabolizing the syndemic, participants reflected on their personal histories as part of a broader reflection on their identities and roles. Many contextualized the COVID-19 pandemic and social justice activism within prior experiences, “like another verse of a song that I’d experienced or heard before,” citing the AIDS epidemic, Hurricane Katrina, or Pearl Harbor as guiding analogies. Participants also connected community responses and activism to events familiar to their families or communities: local school shootings, community suicide clusters, regular hurricane seasons, and activist spirit passed down through generations. These histories encouraged reflection on one’s roles and identities, since, as one participant stated, crises make you “revisit or rethink your priorities and your values; who we are now.”

One such revisitation included the ethical charge of physicians and the role of that identity for each participant: What does it mean to be a doctor during a pandemic? We identified a strong sense of duty and responsibility to the public, despite personal risk, as strongly tied to a physician’s job description: “I was surprised by how quickly people shed their identity as a physician, to hunker down and worry only about themselves and their family. This is a national crisis, you’re a physician, you’re expected to play a role in helping people.” Participants also underscored the importance of “going in,” continuing to work in the hospital despite infection risk and advances in telehealth: “You just can’t be a doctor taking care of patients in this setting without coming in.” Despite this commitment to physician duty, participants overwhelmingly refused to claim the “healthcare hero” narrative, with one stating that “I’ve been careful not to lump myself in with that group because I just haven’t taken those same risks.” Many attributed this difference to working in pediatrics and behavioral health, stating that “as psychiatrists, we are not true COVID-19 front-liners,” downplaying their own contributions in relation to other specialties like emergency medicine.

The question of physician identity seemed a particularly provocative one for psychiatrists, who felt that initially, “our nation had called us to do something very, very different, which we hadn’t been trained for.” Participants felt capable of answering the psychological demands of the pandemic, but some felt helpless in the face of an infectious disease crisis, noting how in the early days, “the psychological impact hadn’t fully hit yet. We didn’t have a major role then.” Some expressed a particular sensitivity to this worry, given the stereotype of psychiatrists as too far removed from the rest of medicine: “We have worked hard to be seen as physicians, … [to have] a bidirectional sense of mutual respect.” Yet as the pandemic progressed, participants emphasized that psychiatrists are “uniquely trained for this COVID-19 moment,” becoming frontrunners in telehealth adoption and tending to the psychosocial impacts of the national crisis. One training director told new trainees, “You are all physicians. You know how trauma and distress impact human functioning. You have the perfect training.”

Alongside a meditation on one’s duty as a physician, participants considered how much of their own identity was ascribed to that role: “How much of you as a person or identity is being a doctor… and how much are you allowed, as a human being, to take care of yourself, separate from being a doctor?” The question of coexisting roles surfaced in discussions of work-life balance (or life-life balance, as one participant put it), as well as discussions of how to best apply one’s expertise. Given the shortage of CAP providers, one participant touched on the difficult decision of taking non-clinical roles given potential “obligations… of being available to people.” Another participant countered, “I’m not naïve. I don’t think that the best use of [my] 40 hours a week is seeing patient after patient” at the expense other roles in research, education, and leadership.

Within meditations on work-life balance, multiple participants reflected on their family lives. In light of the uncertainties and revelations of the pandemic, one early career participant reconsidered their past decision to postpone having a child due to the demands of work; now, they have decided to try to start a family. For others, increased time at home with kids completing online school offered new perspectives, such as one participant struck by the image of their son, home from college, in the backyard: “He used to play in that yard, and I used to look from that window… and here it was, he was home again under very different circumstances.” Moments of novel, unexpected visibility into a new phase of their children’s lives were at times bittersweet, but also motivating and humbling. One participant discovered their son manufacturing plastic face shields for essential workers in the basement, sparking the reflection that “among these unsung heroes… are the kids, who are going to be leading us, not the other way around.” Participants also grappled with supporting family members virtually, such as siblings, parents, and grandparents, through safety concerns and isolation, as well as navigating losses and illnesses from afar.

Among the acute adaptations of roles and identities during the pandemic, several participants focused more intentionally on leadership roles to support colleagues and trainees. For many, shifts in leadership style were forged by the national confrontation with racism and the emotional conversations that followed, with participants adjusting their leadership style according to their own identity. A clinician of color reflected on assuming a facilitator-like leadership stance, acknowledging that “you have to take a different perspective, one that’s more educational; and many times, not one that’s necessarily sharing your own experiences.” The same clinician also noted the need to identify new leaders among colleagues to lead these psychologically heavy conversations about racism and bias in psychiatry, because “it’s so important that it cannot be the responsibility of every African American person to do all the education.”

Participants also emphasized the role of supporting colleagues’ wellbeing, noting the importance of caring for the carers. Many participants created or volunteered for peer support systems, wellness programs, and mental health first-aid for their peers in and beyond psychiatry, providing the additional benefit of increasing gratitude. Regarding mental health care, several participants also noted their own involvement in therapy and the importance of taking time to process the psychological effects of the pandemic. As for social support, renewed investment in social strengthening soothed a “hunger for interactions,” including more frequent check-ins with friends and family, restructured e-meetings that replicated in-person small-talk, and a deeper level of intimacy in previously casual conversations. Some participants also spoke of missing the “random” encounters of an in-person workplace and intentionally building back “incidental” work contacts, setting aside dedicated time to catch up with colleagues who were part of their daily work life despite not sharing any projects or teams.

### *Reimagination:* Who will we become?

In our third domain, “Reimagination,” participants sought for “things to be shaken up and reinvented.” As participants looked forward and wondered, *who will we become?*, we organized participants’ hopes and hesitations into two themes: 1) renewal and recommitment; and 2) psychiatry as advocacy.

#### Renewal and recommitment

Although the syndemic placed additional demands on participants, many also noted that the year had been a very “activating time” for causes they cared about, allowing participants to restructure how they hoped to spend their time moving forward. “With the inspiration and the time, it’s been a fuel,” said one participant of their redoubled efforts against discrimination on their unit. We identified a sense of “returned time”, afforded by the elimination of the daily commute and the flexibility of virtual work. One participant marveled at the gift of walking in nature during the workday, thanks to the “space and flexibility that telepsychiatry allows, that being in person as a physician never really allowed for me.” Returned time cultivated renewed capacity, with participants digging deeper into activism, international mental healthcare volunteering, and research projects with the “energy now that I collected while being able to work from home.” Though optimism was tempered by the losses of the syndemic, several participants expressed hope for the opportunities to move forward, such as in new projects: “They’re literally exploding, and it’s exciting, and it’s thrilling, and it’s going places.”

Another source of energy for many participants was renewed gratitude for colleagues. Despite the initial challenges of intraprofessional trust experienced by some participants, the overwhelming parting message was one of appreciation for others’ hard work. “I want to say thank you to colleagues who have been on the front lines,” said one participant, “for colleagues who have been really supportive of each other during this time and supportive of the patients and families that we work with.” Appreciation extended not just to colleagues in psychiatry, but also to nursing staff and other essential workers. The renewed gratitude provided not only a positive reframing of the pandemic’s difficulties, but also as a recommitment to the profession:

“Hope, privilege, gratitude… those are kind of platitudes, but they’re real. If people can constantly be oriented to this idea that, ‘I am so lucky to be able to do the work that I’m doing, because every single day, I can have an impact on somebody, and I can have an encounter that I experience as meaningful, and important, and helpful to somebody,’ there are very, very few jobs like that.”

Participants also offered specific post-COVID-19 hopes for operational and patient care improvements. Multiple participants hoped for organized pushback against insurance companies’ desire to roll back telehealth affordances, arguing that continued use of telehealth could sustain the increased access to care afforded to many patients, and provide innovative care. Several imagined CAP’s seating “at the head of the table” for the evolution of telehealth, given the field’s success in the early days of the pandemic. In the reimagining of psychiatry’s next steps, we identified a common thread of creativity, with many desiring innovative adjustments to existing practices. Examples included more collaborative virtual teaching (“sharing our crayons”) and utilizing technology to deepen connections (such as in-school observations used as a therapeutic and teaching tool for families, e-consultation models to distribute psychiatric resources more equitably and effectively, and more personal hybrid graduation celebrations). The aperture for creativity also opened a deeper concern, with some participants fearing the perilous potential for unregulated, for-profit telemedicine, or an inevitable return to the status quo.

Within reimagining, we identified a subtheme of recommitment to the profession and its purpose. Some participants reflected on their “origin story” in psychiatry, with several recalling their motivation as wanting to address disparities, to “make things better for people who have, for many reasons, limited access to the resources they need.” Consequently, some desired for CAPs to both serve as more prominent leaders and collaborators and become liaisons not just to children’s health but to families’ wellbeing, “because we really have the capacity to understand how relationships build brains, how society and social determinants of health have such an enormous impact on human functioning.” Several participants also hoped to “banish the term ‘med check,’” finding it an insufficient descriptor of CAP’s deeply interpersonal work: “The best kept secret is, we don’t prescribe as bots…Your story matters to me.”

#### Psychiatry as advocacy

As the syndemic emerged, multiple participants identified the field as being at “a critical crossroads,” with COVID-19 and renewed confrontations with system racism as “accelerants,” as forces “pushing” the field to reckon with existing issues and adapt to necessary change. One such tension is balancing psychiatry’s complicity in and response to systemic racism, with the view of the field as a profession of advocacy. As participants looked toward the future of CAP, we identified conflicting assessments of the field’s ongoing social justice work, and divergent mindsets toward the hope for lasting change.

Increased recognition of the Black Lives Matter movement created space for renewed activism and dialogue between colleagues. One Black participant noted a slight shift in the burden of initiating emotionally heavy conversations about racism, remarking that the shared horrific experience of George Floyd’s death opened an overdue dialogue that more directly acknowledged racism:

“It was also important for people to say it because for the people who have been victims of these experiences for many years, the response has always been, ‘Well, why do you think it’s racism? Or why do you think that’s what it is?’ And to have others recognize how horrible it was without having to be the one to say it, I think was really important.”

Multiple participants described workplace participation in White Coats for Black Lives demonstrations, formation of diversity, equity and inclusion (DEI) committees, and increased activity of identity-based caucuses. Several training directors emphasized the role of social justice education in their residency and fellowship curricula. Attitudes towards professional organizations’ responses differed, with some participants appreciating the immediate strong condemnation and others feeling dissatisfied by the lack of concrete action.

Participants grappled with the call to action based on their own identities but overwhelmingly reflected a motivation for change. Several white leaders reflected on the complexity of supporting trainees, colleagues, and communities of color from a position of racial privilege, such as one respondent “trying to be a good leader in that area as a white man.” Another reflected on the existence of collected data on health disparities that had not been explored: “We should have been looking at this.” For multiple Black participants, the call to action was not a new one, but rather an amplification of the work they had engaged in for years: “I don’t want to make it seem like they’re just now noticing. People have been noticing, and people have been experiencing this for a long time.”

Alongside this delayed collective acknowledgement of systemic racism, fear of institutional inertia and loss of focus also interfered with some participants’ hopes for lasting improvement to post-pandemic psychiatry’s role in social justice work, as well as generally. “Is it an opening?” asked one participant. “Sure. Are we going to take advantage of that opening? We’ll see.” For many, faith in valuable change hinged on concrete action. “We don’t just want to say a statement,” said one Black participant of their institution’s response to the Black Lives Matter protests; “We want to do something and *then* say, this is what we’re doing, versus ‘we stand behind diversity.’” Additionally, some participants expressed frustration over the necessity of pandemic catalysts in the first place: “It’s also just a bit ridiculous, that it takes the murder of a man to be filmed for people to reckon with what we’ve known.” Consequently, although the pandemic figured as a “pivot point” or “fulcrum” for growth, disappointment and frustration tempered optimism about the possibility of meaningful change and highlighted the significance of CAP’s follow-through. As one participant encapsulated the overall outlook, “it’s a fertile time for progress in a positive direction; but like anything, only if we choose to do it as such.”

## Discussion

In seeking to understand the impact of the COVID-19 pandemic on child and adolescent psychiatry in the United States, we identified three domains of change across participants: unsettling, adaptation, and reimagination. Encapsulating these three domains, throughout the six-month study span, participants mulled over three implicit questions: Who have we been? Who are we now? Who will we become? The third question became more poignant as the study drew to a close, as participants grappled with the tumultuous summer of protest and calls for change. Participants at every career stage identified discontents and started to draft a vision for an improved practice; and although mindsets to actualization of that vision diverged from skepticism to optimism that is bounded at best, no participant desired a return to the prior status quo. All viewed the pandemic or syndemic as a reckoning that afforded an opportunity to reimagine a better future.

We began our study with the concepts of aperture and exposure, identifying the COVID-19 pandemic as a catalyst that opened portals to new possibilities or re-illuminated that which had been taken for granted, following Arundhati Roy’s description of the “pandemic as portal” [40]. In the process, however, participants’ reflections guided us toward the concept of pivot points, an evolution allowed for by the inductive approach of thematic analysis [37]: “It’s a pivot moment. It’s a fulcrum,” said one participant of the year’s difficult circumstances. Instead of perseverating on a portal or revelation, participants concerned themselves more with future-forward momentum, taking the unsettling of the syndemic as a prompt to recalibrate the direction of future forces, to consider where to throw one’s weight in order to tip the scale towards progress.

### Opportunities and hesitations

In response to a traumatic year, this momentum-oriented mindset represents an adaptive response that seems promising for the hope of a successful pivot. Olson et al call for pandemic-driven *posttraumatic growth* in healthcare, seeking “positive psychological change experienced as a result of a struggle with highly challenging life circumstances” in a way that creates a “new normal” rather than seeking to return to the status quo [41]. As one participant presciently quipped, “paradigms break, morph, shift when they no longer work for the environment in which they were developed.” In our study, participants’ desires for adaptation and improvement do characterize a posttraumatic growth mindset, outlining Olson et al’s steps of deliberate reflection, seeking collaboration and reinvention, recommitting to public service, and highlighting caring and gratitude. Thus, despite the significant challenges of the syndemic, participants are already focusing on opportunities to improve advocacy efforts alongside colleagues in pediatrics, to revamp collaborations with local school districts to provide better school-based mental health care, and to dig deeper into social determinants of health using existing data or a new research emphasis.

Any desire for change and growth, wrought by the unsettling of the syndemic, is not enough to actualize progress. Participants’ bounded optimism reflected fears of institutional inertia, red tape, and actual backwards movement, such as the planned reinstatement of insurance and licensure barriers that had been lifted to alleviate the immediate demands of the telehealth surge. Participants stressed the continual need to “propagate the opening” of the moment to work toward concrete change. That is, to move beyond COVID-19’s “ground zero empiricism” described by Lorraine Daston, American historian of science, in which “moments of radical novelty and the radical uncertainty novelty emits” lead society to observe a phenomenon for the first time [42]. It is not enough to perceive and study the adaptations to this unfolding syndemic, but to mobilize its reimagination. Moreover, not all of the past year’s ground-zero empiricism is universal: Although some participants experienced the surge in the Black Lives Matter movement’s visibility as revelatory and motivating, many others, particularly participants of color, were not observing the impact of systemic racism in CAP anew, but rather clarifying what they had known all along. Recognizing the varied starting points in tackling the consequences of the syndemic will be important in crafting the next steps for CAP.

### The role of leadership

Overcoming these barriers to change and actually propagating pandemic-driven posttraumatic growth will hinge on the ability of leadership in CAP to respond to discontents and hopes like the ones identified in this study. Leaders must demonstrate the flexibility to avoid the sunk-cost fallacy of sticking with the field’s long invested beliefs and practices in favor of pivoting towards reinvigorated priorities. When leadership is placed “on the edge” amid crises, their communities of practice require connection, communication, and collaboration [43], the last of which being of particular importance to participants in our sample. Leadership that encourages collaboration will be critical in addressing the hopes articulated in this study. Namely, to rebuild intraprofessional trust, find efficient telehealth partnerships, and amplify advocacy efforts with other specialties to push for policy changes, new research initiatives, and insurance or licensing regulatory improvements.

Moreover, with the field of CAP facing a workforce shortage [44], leaders in training and education will be even greater importance in recruitment and career development of a new workforce generation, as well as setting the vision statement for “CAP reimagined” and informed post-pandemic. Already, training directors in this study highlighted the importance of social justice education and building a strong sense of community among their virtual cohorts; leaders in education are devising ways to challenge the “hidden curriculum” of medical education to center resiliency and burnout prevention in pandemic response for current residents and fellows [45]; and residents are outlining next steps for resident education, such as identifying a program’s core values [46], and highlighting the benefits of a Triple Board education [47].

### Limitations and future directions

We recognize several limitations in our sample and approach. First, although we included clinicians across the career development spectrum, our sample was weighted toward senior clinicians 20 or more years past CAP graduation. The resulting emphasis on the importance of leadership may therefore reflect the sample’s seniority and position, with half of the sample serving as either division chiefs or training program directors. Although participants from all levels of practice did give a comprehensive account of team dynamics and echoed the importance of refining values and priorities, our insights could be deepened further with future studies including a wider range of perspectives; in particular, those of trainees like residents, fellows, and perhaps medical students invested in CAP, as well as those of non-physician behavioral health specialists within CAP. Second, although this study aimed to “develop the film” of participants’ photo reflections, we recognize that this reflective exercise occurred in the midst of the syndemic, rather than serving as a retrospective analysis of completed events. In describing the opportunities for posttraumatic growth, Olson et al caution against pressuring individuals to seek growth too soon after a traumatic event ends, let alone during a crisis [41]. Although the qualitative nature of this study offered a moment of reflective stillness for which several participants expressed gratitude, the study’s close proximity to the daily events of the syndemic undoubtedly influenced the nature of participants’ commentary. A follow-up study exploring clinician’s takeaways once the field is fully in the recovery phase would be a valuable complement to our investigation.

## Conclusion

A qualitative exploration of child and adolescent psychiatrist’s responses to the COVID-19 pandemic revealed a tentative yet growth-oriented reimagining of the field in response to unearthed discontents, as well as a reappreciation of the field’s strengths and a recommitment to a profession of advocacy. In the throes of a national syndemic, clinicians pondered several questions: Who have we been? Who are we now? Who will we become? Now, with the COVID-19 pandemic raging into its second year, a fourth question arises: How can we facilitate the passage from a time of crisis to our reimagined future?

## Supplementary Material

Supplement

Supplement

## Figures and Tables

**Table 1.** *Unsettling:* themes and sample quotes

Theme	Sample quote
Daily workplace inefficiencies	If you look at the proportion of humans working in healthcare, and split them up into clinicians versus administrators, there’s a really cool graph…where you see the administrative part grow like this, and the provider or the clinician piece doesn’t grow as much. I want to leave that behind. Meetings where people need to have lots of meetings to justify their time and justify why they exist in an organization, these layers of bureaucracy and committees and task forces where it feels like things are made more difficult for pointless reasons. I guess the best way to frame it is I want to leave behind all the side effects that go along with that chart.
Barriers to patient care	It was just amazing how in two weeks everything was available. Insurance companies are going to cover everything. All of a sudden, we have institution-wide availability of platforms to be able to connect with our patients and there’s all of a sudden this flexibility that we have in being able to connect with other family members, who we couldn’t connect with before.
Intraprofessional devaluation and loss of trust	I’m not a big sour grapes kind of guy, but there’s something that I’m clearly dealing with. I would like to tell my colleagues in child psychiatry: “talk less, do more.” I’m not preaching this to the world of child psychiatry at large because I see so many examples of how incredibly they have done. But when I see…the roster of who’s on call, which is one very concrete way, it’s like talk less, do more. That’s the bitter taste.

**Table 2.** *Adaptation:* themes and sample quotes

Theme	Sample quote
Operational adjustments	If I’m ever having a session and I have to do it from the car, well, there’s a boundary around it. It’s a confidential space, no one can see or hear you, you can see I’m in my car. So the patient feels comfortable that no one can hear you, but you’ve had to set up your office there while waiting to pick up your son and his two friends from a carpool after baseball practice. That’s a real-life example of how you have to integrate your professional and personal responsibilities.
Affordances and limitations of virtual work	I was having an intake with a patient…and then the mother and then the father came… Then the grandparents sat here like this, as if they’re watching a movie. This is not a movie…The grandparents were mostly there and saying, “Yes, that’s true. That’s true. She does that a lot,” but it was like they were watching a movie.
Evolution of personal and professional identity	I’ve had communication with people that I work with that I’ve never had before. Learned things about them, they’ve learned things about me that have been completely eye-opening, and I think have brought us closer together in a lot of ways. I don’t think it would have been possible if COVID never existed. Certainly, I have been doing more connecting with people who I hadn’t been connecting with on a regular basis. Some of those are people who have nothing to do with child psychiatry, but who are incredibly central to my wellbeing and humanness.

**Table 3.** *Reimagination:* themes and sample quotes

Theme	Sample quote
Renewal and recommitment	I really appreciate the reduction in silos. The pandemic did help us make progress by saying, “we’re all one team and if we find a solution, we should share the solution, we should help each other.” So I feel like leaving behind the silos and really seeing… child psychiatry across the state, across the nation, all as one family which has resources and ideas that could be shared.
Psychiatry as advocacy	I hope that people are understanding as the world diversifies and becomes more global… that all of these things interdigitate with one another, and that you really can’t unpack them unless you understand them… If you don’t understand a person’s context, you cannot possibly understand their disease… Have you walked down that neighborhood? There are no grocery stores, there actually are no green spaces, there actually are no sidewalks. So that is intrinsically related to people’s health. There is no broadband, there is a lot of noise, there’s toxic fumes in the air, these buildings are old… And to not understand how all of that impacts the child’s health is to negate what it is to be a physician. Our biopsychosocial formulation is supposed to get at that. But I don’t think that we do it in a way in which is nuanced enough to understand how social determinants of health are based on these structural systems.
