# Supplementary material for: Pivoting in the pandemic: a qualitative study of child and adolescent psychiatrists in the times of COVID-19
Source: Res Sq. 2021 Mar 4:rs.3.rs-287057. Preprint. [Version 1] doi: 10.21203/rs.3.rs-287057/v1 (PMC7941638; doi:10.21203/rs.3.rs-287057/v1)
Supplement: Supplement [file e0b6a5d7942562365190b5ad.docx]

| **Appendix 2.  Characteristics of study participants (n = 24)** |  |  |
| --- | --- | --- |
|  |  |  |
| **Category** | **n** | **%** |
| Sex |  |  |
| Female | 12 | 50 |
| Male | 12 | 50 |
| Ancestry |  |  |
| Asian | 3 | 13 |
| Black | 6 | 25 |
| Latinx | 6 | 25 |
| White | 15 | 63 |
| Affiliation |  |  |
| IMG | 5 | 21 |
| LGBTQ | 2 | 8 |
| US Region |  |  |
| Northeast / Midwest | 10 | 50 |
| South | 5 | 21 |
| West | 7 | 29 |
| Career stage (years since CAP graduation) |  |  |
| Early (<10) | 5 | 21 |
| Mid (10 - 20) | 5 | 21 |
| Senior (>20) | 14 | 58 |
| Faculty appointment |  |  |
| Primary / ladder | 19 | 79 |
| Clinical / adjunct | 5 | 21 |
| Administrative role |  |  |
| Division chief | 6 | 25 |
| Training program director | 6 | 25 |
|  |  |  |
| *Note:* IMG = international medical graduate. |  |  |
